# COLLABORATION IN CARING FOR OLDER ADULTS WITH DEMENTIA LIVING IN LONG-TERM CARE FACILITIES

**DOI:** 10.1093/geroni/igad104.2744

**Published:** 2023-12-21

**Authors:** Phatt Thaitrong, Joy Douglas, Linda L Knol, Amy Ellis, Hyunjin Noh, Shena Sanchez

**Affiliations:** The University of Alabama, Tuscaloosa, Alabama, United States; The University of Alabama, Tuscaloosa, Alabama, United States; The University of Alabama, Tuscaloosa, Alabama, United States; The University of Alabama, Tuscaloosa, Alabama, United States; University of Alabama, Tuscaloosa, Alabama, United States; The University of Alabama, Tuscaloosa, Alabama, United States

## Abstract

Current evidence calls for an interdisciplinary, team approach to caring for older adults with dementia. Registered dietitian nutritionists (RDNs) are interdisciplinary team members who play an important role in nutrition and dementia care. Our study explored how RDNs collaborate with licensed healthcare providers and direct care staff to provide nutrition care for older adults with dementia living in long-term care (LTC) facilities. RDNs who work with residents with dementia in the U.S. (n = 18) were recruited to participate in one of five focus groups, where they described the various disciplines they collaborate with. Focus groups were led by a trained moderator using a semi-structured interview guide, transcribed verbatim, and audited by the research team for accuracy. Transcripts underwent descriptive content analyses using NVivo© software. Descriptive content analyses revealed the following diverse groups who work collaboratively with RDNs: 1) nursing staff, 2) foodservice staff, 3) therapists, 4) physicians, and 5) other non-clinical staff, including activities staff and nursing home administrators. Additionally, RDNs reported they worked closely with residents’ family members and resident members of the food committee. In conclusion, in LTC settings, RDNs collaborate with various groups, encompassing clinical, non-clinical, and even residents/family to care for older adults with dementia. However, collaboration is not limited to clinical staff. To provide optimal nutrition care for older adults with dementia, it is important to recognize the value of collaborating with both clinical and non-clinical stakeholders to meet resident needs.

